# Language Dysfunction in Schizophrenia: Assessing Neural Tracking to Characterize the Underlying Disorder(s)?

**DOI:** 10.3389/fnins.2021.640502

**Published:** 2021-02-22

**Authors:** Lars Meyer, Peter Lakatos, Yifei He

**Affiliations:** ^1^Research Group Language Cycles, Max Planck Institute for Human Cognitive and Brain Sciences, Leipzig, Germany; ^2^Clinic for Phoniatrics and Pedaudiology, University Hospital Münster, Münster, Germany; ^3^Center for Biomedical Imaging and Neuromodulation, Nathan Kline Institute, Orangeburg, NY, United States; ^4^Department of Psychiatry and Psychotherapy, Philipps-University Marburg, Marburg, Germany

**Keywords:** neural tracking, neural oscillations, schizophrenia, electroencephalography, language comprehension, speech perception

## Abstract

Deficits in language production and comprehension are characteristic of schizophrenia. To date, it remains unclear whether these deficits arise from dysfunctional linguistic knowledge, or dysfunctional predictions derived from the linguistic context. Alternatively, the deficits could be a result of dysfunctional neural tracking of auditory information resulting in decreased auditory information fidelity and even distorted information. Here, we discuss possible ways for clinical neuroscientists to employ neural tracking methodology to independently characterize deficiencies on the auditory–sensory and abstract linguistic levels. This might lead to a mechanistic understanding of the deficits underlying language related disorder(s) in schizophrenia. We propose to combine naturalistic stimulation, measures of speech–brain synchronization, and computational modeling of abstract linguistic knowledge and predictions. These independent but likely interacting assessments may be exploited for an objective and differential diagnosis of schizophrenia, as well as a better understanding of the disorder on the functional level—illustrating the potential of neural tracking methodology as translational tool in a range of psychotic populations.

## Introduction

Schizophrenia is characterized by language deficits ranging from lower acoustic and phonetic levels to higher semantic and syntactic levels that are highly functionally relevant (Bleuler, 1950; Chaika, 1990; DeLisi, 2001; Covington et al., 2005). Among others, key features of patients’ speech include flattened prosody, simplified syntax, and loosened semantic associations (for review, see Andreasen, 1979; Kircher et al., 2014). In comprehension, correspondingly, patients are impaired in the processing of linguistic information at these levels (Leitman et al., 2005; Mohammad and DeLisi, 2013; Javitt and Sweet, 2015; Moro et al., 2015). In addition, major symptoms of schizophrenia such as auditory hallucinations and delusions are thought to be closely related to impaired speech perception and language comprehension (Brown and Kuperberg, 2015). To date, the neuropathology of language impairments in schizophrenia remains unclear (Miller and Isard, 1963; Morice and Delahunty, 1996; Crow, 1998; DeLisi, 2001; Bagner et al., 2003; Li et al., 2009; Brown and Kuperberg, 2015; Hirano et al., 2020). In this paper, we suggest that the understanding of language deficits in schizophrenia could benefit from analyzing neural oscillations with neural tracking methodology. Oscillations can be aligned to speech and this alignment seems to be guided by attention, especially in “cocktail party” settings (e.g., Vander Ghinst et al., 2016; for review, see Lakatos et al., 2019a)^1^. Therefore, we propose that multi-scale (on phrasal/syllable temporal scales) oscillatory alignment provides a novel tool for assessing language dysfunctions on various linguistic levels.

Our Hypothesis and Theory article considers three main issues: In the first part, we discuss possible ways to address deficits in auditory perception and speech tracking as such. In the second part, we delineate probable relationships between prosodic–syntactic deficits and altered delta-band oscillations. In the third part, we hypothesize that impaired semantics in schizophrenia could result from altered beta–gamma coupling. In the last section, we discuss how to pursue these hypotheses by combining naturalistic experimental paradigms with methodology that assesses the exogenous neural tracking of auditory–phonetic information and the endogenous generation of abstract linguistic information.

### Auditory Perception and Speech Tracking: Impaired Theta-Band Oscillations

Deficits in auditory processing could underlie a range of language-related symptoms in schizophrenia (Javitt and Freedman, 2015). Such deficits do not include hearing *per se* (McKay et al., 2000; Javitt, 2009), as detection thresholds, sensitivity to loudness, spatial localization, and the P1/N1 complex in the event-related brain potential (ERP) for isolated sounds are all intact (Javitt and Freedman, 2015). Yet, patients show reduced auditory mismatch negativities (MMN; for review, see Näätänen and Kähkönen, 2008; Todd et al., 2013; Michie et al., 2016) to both speech and non-speech sounds (Kasai et al., 2002, 2003). In addition, P1/N1 difference waves are altered under repetition priming and sensory gating (Freedman et al., 1987; Adler et al., 1998; Patterson et al., 2008), and for perception of words differing in lexicality (Hirano et al., 2008). Moreover, the N1 difference between self-produced and presented auditory stimuli has been found abnormal in experiments investigating potential deficits in corollary discharge and efference copy during auditory speech processing (Ford et al., 2001, 2007a, b). Apart from the MMN, P1, and N1 components, a reduction of the P300 in oddball paradigms is a robust neural marker of schizophrenia (Ford et al., 1994, 2008; Higashima et al., 2003). In sum, despite the fact that isolated sounds appear to be processed normally in schizophrenia, earlier behavioral and ERP evidence suggests that patients with schizophrenia are impaired in a range of auditory processes—whenever patient’s perception of tones or speech involves top-down influences—thus suggesting predictive coding rather than audition impairments in schizophrenia (Adams et al., 2013; Sterzer et al., 2018; Howes et al., 2020; Smith et al., 2021).

In spite of these rather robust results, the altered difference ERPs calculated from stimulus train of simple tones or speech sounds (i.e., MMN and P300) are hard to dissociate from altered oscillatory activity due to the fact that oscillatory phase reset contributes heavily to ERPs (Klimesch et al., 2007; Ding and Simon, 2014; Obleser and Kayser, 2019; Haegens, 2020). It has been argued that the MMN represents a mainly theta-band phase reset that occurs in the extragranular layers of the auditory cortex; moreover, the MMN is vulnerable to blockage of the N-Methyl-D-Aspartate Receptor (Lakatos et al., 2019b). NMDA receptor related deficits are hypothesized to underlie a range of symptoms as well as auditory deficits in schizophrenia (Kort et al., 2017; Corlett et al., 2018; Javitt et al., 2020). In addition, altered P300 responses in schizophrenia were observed in parallel with altered theta-band oscillations (Ford et al., 2008). In sum, this initial evidence leaves it open whether aberrant theta oscillations are impaired independently of evoked responses as shown in the ERPs, and thus they both might contribute to auditory and language deficits in schizophrenia.

Here, we propose that neural tracking methodology that employs naturalistics paradigms should allow researchers and clinicians to better focus on theta-band oscillations without the confounding ERP alterations elicited by controlled experiments. In particular, this contrasts with oddball experiments and the associated MMN and P300 alterations. Theta-band oscillations are thought to phase-lock to the acoustic edges of syllables, aiding their segmentation or even identification (Luo and Poeppel, 2007; Howard and Poeppel, 2012; Gross et al., 2013; Peelle et al., 2013; Doelling et al., 2014). Furthermore, processing of phonemes in context (e.g., labeling of phonetic features, predicting the likelihood of upcoming phonemes) can be investigated together with envelope tracking with state-of-the-art multivariate analysis techniques, by close examination of low-frequency oscillations including the theta band (e.g., Di Liberto et al., 2015, 2019; Daube et al., 2019) and the delta band (see below). To date, however, only few recent studies have reported altered theta-band power and phase aberrence in schizophrenia, which used simple tones delivered as part of traditional oddball and gating paradigms (Lakatos et al., 2013; Kantrowitz et al., 2016; Lee et al., 2017). Thus, investigating potential impairments of theta-band oscillations during neural tracking of naturalistic speech could contribute to extant research, and could provide an unique window into understanding the neuropathology of language deficits in schizophrenia. Further potential of the combined use of computational modeling and neural tracking methodology to dissociate acoustic, phonological, and linguistic processing is provided below.

### Prosody and Syntax: Abnormal Delta-Band Oscillations?

Auditory processing deficits in schizophrenia are considered as reflecting “up-stream” functions, such as prosody (Javitt, 2009). Flattened prosody in production is a negative symptom of schizophrenia, characterized by reduced modulation of fundamental frequency and amplitude, utterances that are shortened and less variable in duration, and pauses that are longer and more variable (Alpert et al., 1989, 2000; Covington et al., 2005). Such language production related indices can classify schizophrenia incidence with high accuracy (Püschel et al., 1998; Rapcan et al., 2010; Martínez-Sánchez et al., 2015) and may help to detect risk (Cibelli et al., 2017). In comprehension, patients struggle to infer emotions and communicative intentions from prosody (e.g., Pawełczyk et al., 2018a). While this is sometimes discussed as epiphenomenal to impaired emotion reception (Murphy and Cutting, 1990; Mitchell and Crow, 2005; Hoekert et al., 2007; Lin et al., 2018), flattened prosody occurs also when emotional vocabulary is intact (Alpert et al., 2000). Moreover, auditory deficits in prosody perception predict most variance associated with impaired comprehension of emotional prosody (Leitman et al., 2005; Dondé et al., 2017), and the inference of emotion is improved by prosody training (Lado-Codesido et al., 2019).

In addition to prosody, syntactic impairments have been observed. Syntactic rules serve to decode the propositional relationships amongst words in speech (cf. Martin, 2020). Patients with schizophrenia do not reliably detect syntactic errors (Moro et al., 2015), and their working memory benefits less from syntactic structure (i.e., no sentence superiority effect; Bonhage et al., 2017; Li et al., 2018). Alternatively, syntactic rules might be intact, but their top-down influence on perception is temporally distorted (Rochester et al., 1973). In healthy populations, syntactic boundaries (e.g., clause endings) influence perception, such that acoustic events that are experimentally displaced from a boundary are perceptually “dragged toward it” (Fodor and Bever, 1965). This effect appears to be altered in patients (Rochester et al., 1973). A temporal deficit would be also consistent with reports of turn-taking deficits (Sichlinger et al., 2019). Healthy speakers tend to indicate turn-giving with prosodic markings (Levinson, 2016), but patients often fail to do so (Bellani et al., 2009; Colle et al., 2013; Pawełczyk et al., 2018b). In particular, the timing of turn-giving is affected, such that variance in utterance duration decreases and variance in pause duration increases (Alpert et al., 2000).

**FIGURE 1:**
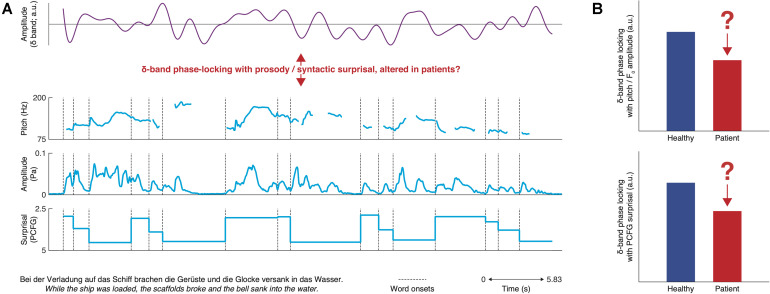
Overview of hypotheses for prosodic and syntactic deficits. **(A)** top: synthetic delta-band oscillation; bottom: frequency– and amplitude modulations corresponding to the pitch track as well as computational-linguistic measures of the application of syntactic rules for the example sentence at the bottom. It is hypothesized that delta-band phase-locking is impaired in patients with schizophrenia and that depending on the underlying disorder, this abnormality could be restricted to either prosody or syntax. For demonstration, a strongly phase-locked delta-band oscillation was generated *via* a randomization procedure. **(B)** Hypotheses as bar charts: depending on the underlying deficit, either phase-locking to prosody or syntax should be impaired in patients.

We propose that the respective neural counterparts of prosodic and syntactic symptoms could be assessed by focusing on delta-band oscillations (i.e., 0.5–4 Hz; Buzsaki, 2006; Güntekin and Başar, 2016; Figure 1), as the time scale of these neural oscillations can be clearly linked to speech structure (Giraud and Poeppel, 2012; Ding et al., 2016). While delta-band oscillations are certainly altered in schizophrenia (for review, see Başar and Güntekin, 2008; Ford et al., 2008; Doege et al., 2010; Lakatos et al., 2013), there is no unitary link with the above symptoms yet. We propose that establishing such a link would support differential diagnosis of the underlying disorder, and it could also contribute to the ongoing struggle for a dissociation of the functional roles of delta-band oscillations in prosody, syntax, and timing (Lakatos et al., 2008; Ghitza, 2017; Meyer et al., 2017, 2019).

On the one hand, in healthy subjects, delta-band oscillations synchronize with prosody (Bourguignon et al., 2013; Gross et al., 2013; Mai et al., 2016; Molinaro et al., 2016), the perception of which is impaired in schizophrenia (e.g., Dondé et al., 2017). On the other hand, delta-band frequencies match the rate of occurrence of syntactic phrases and sentences (Ding et al., 2016) and delta-band phases are aligned to syntactic structure (Brennan and Martin, 2020) and information content (Meyer and Gumbert, 2018), independently to prosody (Meyer et al., 2017). Healthy subjects show increased delta-band power during working memory encoding of syntactically structured relative to unstructured word sequences (Bonhage et al., 2017). In contrast, working memory encoding in schizophrenia patients does not benefit much from syntactic structure (Li et al., 2018).

Instead of prosodic and syntactic deficits as such, abnormal delta-band oscillations could also indicate an underlying timing deficit. Lakatos et al. (2013) observed reduced delta-band phase alignment (measured by inter-trial phase coherence) in patients across the isochronous trials of an auditory oddball experiment. The authors interpret this as indicating deficient temporal prediction, mediated by the alignment of oscillatory brain activity to external stimulus timing (i.e., oscillatory entrainment). They also demonstrate that the lack of phase alignment is associated with reduced behavioral performance and correlates with clinical symptoms. This interpretation of their results stems from prior work related to the role of delta-band oscillations in temporal prediction (Lakatos et al., 2008; Stefanics et al., 2010; Arnal et al., 2014; Breska and Deouell, 2017; Jones et al., 2017; Rimmele et al., 2018; Donhauser and Baillet, 2020). Specifically, Stefanics et al. (2010) observed enhanced auditory target detection during specific phase intervals that were elicited through prior rhythmic stimulation (cf. Henry and Obleser, 2012; Hickok et al., 2015). Delta-band phase is an imprint of the neuronal excitability of auditory regions (e.g., Lakatos et al., 2008). Delta-band oscillations could thus likely serve prediction by preallocating excitability and functional connectivity within relevant brain circuits to the expected onsets of upcoming stimuli (e.g., Lakatos et al., 2008, 2009). Our recent results speak in favor of a link between the involvement of the delta band in syntactic processing and its involvement in temporal prediction. We found that the delta phase is not just generally correlated with syntax, but it is more specifically correlated with the strength of syntactic predictions (Hale, 2001; Levy, 2008; Meyer and Gumbert, 2018).

### Semantics: Impaired Predictive Coding in the Beta- and Gamma-Bands?

Semantic impairments in schizophrenia are less controversial than prosodic and syntactic impairments. Patients commonly display hyperactivation of lexical-semantic associations. While healthy individuals associate *lion* with *tiger* but not with *stripes*, patients with schizophrenia may do so. Accordingly, patients produce words that are less directly related to their intended message (Bleuler, 1950). For example, they might complain about their chest pain by saying *I wonder if my box is broken* (Chaika, 1990). Correspondingly in comprehension, patients show enhanced semantic priming effects (Spitzer et al., 1994; Weisbrod et al., 1998; Kreher et al., 2009). However, depending on the task under study, patients may also exhibit a more restricted semantic network than healthy subjects during comprehension (Kreher et al., 2009). The comprehension deficits manifest beyond the word level, that is, real-world objects and events are commonly associated with special and negative meaning, a defining feature of delusions.

Most electrophysiological literature on semantic comprehension deficits in schizophrenia has exploited the N400 component of the evoked response (ERP), typically manipulating the semantic/discourse fit between a target word and its preceding word/sentence context (Kutas and Hillyard, 1980; Hagoort et al., 2004; Nieuwland and Van Berkum, 2006; Lau et al., 2008; Kutas and Federmeier, 2011). These studies have offered valuable insights into how word– and sentence-level semantics are disrupted vs. preserved in schizophrenia (Mohammad and DeLisi, 2013; Kiang and Gerritsen, 2019).

Word-level semantic processing in schizophrenia is most commonly investigated *via* priming paradigms. Yet, the literature is inconsistent in terms of whether the priming-N400 effects are enhanced or reduced in patients (Mathalon et al., 2002, 2010; Salisbury, 2008; Kuperberg et al., 2019; Sharpe et al., 2020). This discrepancy may result from impairments at different levels of the linguistic hierarchy. Patients may be impaired in lexical access (Kuperberg et al., 2019), may suffer from reduced or enhanced semantic activation (Titone et al., 2000; Mathalon et al., 2010), or may fail to derive predictions from the word context (Sharpe et al., 2020). Of note, the prediction failure account accords with results from sentence-level N400 studies: Whereas the N400 reflecting semantic retrieval and integration seems to be unaffected in schizophrenia (Kuperberg et al., 2006), converging evidence has shown that patients are unable to utilize contextual information to suppress irrelevant meanings of a target word, for example, when comprehending a homophone (Sitnikova et al., 2002). Hence, it has been proposed that semantic deficits in schizophrenia may originate from a general inability to integrate and update predictions of higher linguistic levels (e.g., context) with lower-level semantic inputs (Brown and Kuperberg, 2015). However, as it remains unclear whether the N400 indexes prediction, prediction error, or a combination of both (Kutas and Federmeier, 2011; Bornkessel-Schlesewsky and Schlesewsky, 2019; He et al., 2020; Kuperberg et al., 2020; Nieuwland et al., 2020), it also remains unresolved whether linguistic prediction or prediction error underlies semantic impairments in schizophrenia.

**FIGURE 2:**
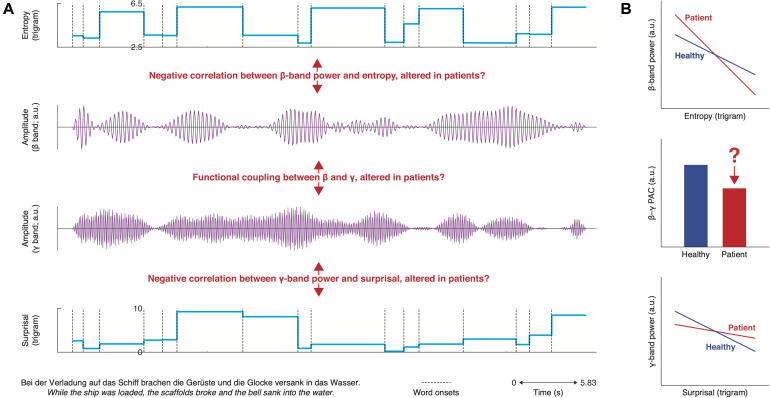
Overview of hypotheses for semantic deficits. **(A)** top: word-by-word entropy time course for example sentence (bottom left); middle: synthetic beta– and gamma-band time courses; bottom: word-by-word surprisal time course for example sentence (bottom left). We hypothesize that the relationship between beta-band power and entropy as well as between gamma-band power and surprisal is abnormal in schizophrenia patients. Additionally, phase–amplitude coupling of the beta and gamma band might be disturbed. These effects will likely differ amongst schizophrenia subgroups (e.g., hallucinators vs. non-hallucinators). **(B)** Summary of hypotheses.

With the equivocal interpretation of N400 alterations in mind, we propose to investigate semantic deficits by examining neural oscillations in the beta– and gamma-bands (for a possible relationship between the N400 and delta-band oscillations, see Roehm et al., 2007). The maintenance of semantic top-down predictions has been associated with beta-band power, whereas gamma-band power reflects the integration with bottom-up semantic input (Lewis and Bastiaansen, 2015; Lewis et al., 2015; Meyer, 2017). In healthy populations, at the semantic level, the sensitivity of beta–gamma-band power has been reported in a series of studies (Hagoort et al., 2004; Wang et al., 2012a, b, 2018; Kielar et al., 2014, 2015). Notably, as the majority of these studies have leveraged the classic semantic violation paradigm, despite a theoretical dissociation, it remains controversial how beta– and gamma-bands map to prediction or prediction error during sentence-level processing (for review, see Prystauka and Lewis, 2019). We thus suggest a naturalistic approach (Figure 2), allowing for the dissociation of prediction and error at the single-word level, as well as an independent comparison between healthy and clinical groups. In healthy participants, beta-band power decreases for more precise prediction; for decreased error, gamma-band power increases accordingly. In addition, when predicted and incoming information match, cross-frequency coupling between the beta and gamma bands would increase (Roopun et al., 2008; e.g., Engel and Fries, 2010; Chao et al., 2018). The beta–gamma interplay offers a promising candidate mechanism that bridges predictive and integrative semantic processes. It also forms the basis for a plausible unifying theory linking predictive deficits in schizophrenia across functional domains outside of language. In the sensory domain, our previous work has shown that gamma power is less modulated for schizophrenia in response to prediction error on the acoustic level (Lakatos et al., 2013). In a similar vein, effects for predictive beta modulation in schizophrenia has been reported when patients are engaged in social interactive games (Billeke et al., 2015). Oscillations across frequency ranges appear to be coupled (Lakatos et al., 2005; Canolty et al., 2006; Canolty and Knight, 2010). Thus, it is worthwhile to investigate the cross-frequency dynamics (e.g., phase–amplitude coupling) in schizophrenia (Kirihara et al., 2012; Hirano et al., 2018) during language processing and speech perception to examine, for example, if impaired beta–gamma oscillations will impact delta oscillatory tracking and vice versa.

A particular focus on dysfunctional beta– and gamma-band oscillations has the additional potential of providing a theoretical explanation of core symptoms of schizophrenia, such as auditory hallucinations and delusions, and on how these symptoms, in turn, impact upon sensory tracking and linguistic prediction. Impairments of prediction in schizophrenia can be *nuanced (Sterzer et al., 2018)*: it has been proposed that auditory hallucinations may derive from overly precise (stronger) prediction (Corlett et al., 2018; Heinz et al., 2019), whereas delusions are related to imprecise (weaker) prediction (Stuke et al., 2018), even if both symptoms often co-occur. We propose that physiologically, hallucinations and delusions are perpetrated by stronger vs. weaker synchronization of brain activity correspondingly in certain frequency bands.

More importantly, both stronger and weaker predictions may occur at hierarchically different levels of sensory and higher cognitive processes in schizophrenia, and may be subject to interaction across levels (Horga et al., 2014; Teufel et al., 2015; Alderson-Day et al., 2017; Powers et al., 2017), indicating the importance of changes in functional connectivity. In the language domain, word-level priming N400 deficits is proposed to be related to delusion severity, thus may support impaired semantic prediction (Kiang and Gerritsen, 2019). In relation to neural oscillations, it has been reported that prestimulus beta-band phase is inversely related to hallucination severity when patients produce speech and listen to the speech sound that they have produced (Ford et al., 2007b). Although the authors did not report power modulation, this study might be an indication of potential link between auditory hallucinations and the beta-band phase in terms of aberrant prediction across comprehension and production of speech (Wang et al., 2012a; Piai et al., 2014; Lewis et al., 2015). Moreover, the most replicated oscillatory correlate of auditory hallucinations is reported in the literature investigating auditory steady-state responses: Gamma-band (usually 40Hz) power and inter-trial phase coherence has been shown to correlate well with hallucination severity (Spencer et al., 2008; Mulert et al., 2011). Notably, steady-state responses reflect a mixture of stimulus-specific evoked responses and the resonant response of the sensory cortices. Therefore, they may not be interpreted on a par with endogenous gamma oscillations (Duecker et al., 2020). However, the strong correlation between the gamma-band responses and auditory hallucinations, together with the reported beta alterations, suggest that both frequency bands are valuable candidates of evaluating dysfunctional predictive coding from a phenomenological perspective. Apart from semantic processing, recent studies employing naturalistic approaches suggest that auditory processing in the form of speech tracking or phonemic prediction—as reflected by low-frequency oscillations in the theta and delta bands—may be subject to top-down influence such as semantic or contextual prediction (Broderick et al., 2019; Heilbron et al., 2020). These emerging studies are prime examples of how naturalistic approaches might directly contribute to the underlying neuropathology of auditory hallucinations in schizophrenia: Instead of observing generally modulated semantic prediction (beta) and auditory tracking (theta) for non-hallucinating patients, we propose to investigate how these processes are enhanced or reduced in hallucinating patients, and how are the functional coupling between beta-theta bands altered in hallucinations.

### Toward Naturalistic Experiments for Schizophrenia Research

Most electrophysiological studies on language deficits in schizophrenia employed controlled factorial designs that used isolated sentences or word pairs. These studies have provided valuable insights into the neuropathology of schizophrenia, but face limitations. First, repetitive experimental procedures limit ecological validity (Brennan, 2016; Willems et al., 2016; Hamilton and Huth, 2018; Hasson et al., 2018; Kandylaki and Bornkessel-Schlesewsky, 2019; Shamay-Tsoory and Mendelsohn, 2019). Second, the typical isochronous presentation of words and sentences (e.g., oddball paradigms, rapid serial visual presentation, RSVP) triggers sequences of evoked responses that have the potential to mask oscillatory activity (Meyer et al., 2020; Poeppel and Teng, 2020); note that this advantage only pertains to those evoked components that are genuine to oddball designs (e.g., P300, see above) and RSVP designs (e.g., repetitive visual onset responses). Third, factorial subtraction approach (e.g., standard – deviant, congruent – incongruent) does not allow straightforward dissociations between acoustic–phonetic and abstract linguistic processes (e.g., Nieuwland et al., 2020). Finally, it is difficult to measure interactions across linguistic levels with factorial approaches (Brown and Kuperberg, 2015; Sterzer et al., 2018).

We thus propose to address language deficits in schizophrenia with naturalistic experiments using ecologically-valid language stimuli (Hamilton and Huth, 2018; Kandylaki and Bornkessel-Schlesewsky, 2019). In such naturalistic experiments, participants are presented with entire narratives (e.g., Stehwien et al., 2020). This enhances feasibility under the temporal and monetary constraints of clinical research while still increasing statistical power and flexibility beyond factorial designs. Narratives also allow the analysis of neural tracking of acoustic and phonetic modulations at the sampling rate of the electrophysiological recording or phonetic–phonological annotation (e.g., Gross et al., 2013; Bastos and Schoffelen, 2015; Di Liberto et al., 2015; Daube et al., 2019). This in turn allows researchers to directly address the above hypothesis on dysfunctional theta-band tracking that we presented in our first scenario above. In parallel, multiple levels of word-by-word/phrase-by-phrase linguistic processing can be analyzed through domain-specific metrics derived by computational-linguistic modeling (e.g., Hale, 2001, 2016; Levy, 2008; Frank et al., 2015; Brennan, 2016). Emerging studies have approached naturalistic story comprehension to investigate language processing in healthy aging (Broderick et al., 2020; Cuevas et al., 2020). In schizophrenia research, naturalistic experiments were proposed for the study of social dysfunctions (Leong and Schilbach, 2019; Brandi et al., 2020). In the language domain, an eye-tracking study using a visual-world paradigm has looked into the impact of higher-level discourse on ambiguity resolution (Rabagliati et al., 2019). We have recently investigated patient’s processing of multimodal stories (i.e., auditory story, manual gestures) using functional magnetic resonance imaging, showing that manual gestures can enhance patients’ reduced semantic activation in a left fronto-temporal network (Cuevas et al., 2019; Cuevas et al., in preparation).

For the second scenario outlined above, the investigation of impaired syntactic and prosodic processing in schizophrenia, a naturalistic approach would allow for a dissociation of the previously proposed prosodic and syntactic deficits. To approximate prosody, the audio stimulus would be low-pass filtered to yield those frequency modulations that correspond to pitch changes (Meyer et al., 2017; Meyer and Gumbert, 2018). Alternatively, the speech envelope could be low-pass filtered, yielding pitch amplitude modulations (Bourguignon et al., 2013; e.g., Gross et al., 2013; Mai et al., 2016). To concurrently model the application of syntactic rules, computational-linguistic modeling would be employed. In electrophysiological research on healthy populations, parsers that operationalize probabilistic context-free grammars are used frequently (e.g., Roark et al., 2009; Frank et al., 2015; Meyer and Gumbert, 2018; Vassileiou et al., 2018). Such algorithms are trained on large corpora annotated with part-of-speech labels and syntactic structures, enabling subsequent annotation of the narrative used for stimulation. Information theory is then applied to quantify syntactic processing difficulty (Shannon, 1948; Hale, 2001, 2016). The prosodic and syntactic regressors would then be related statistically to the electrophysiological data. For prosody, this could be achieved using a variant of speech–brain coupling methodology (for review, see Bastos and Schoffelen, 2015; Poeppel and Teng, 2020). For syntax, time-resolved multiple regression (Sassenhagen, 2019) or multivariate temporal response functions (mTRF, Crosse et al., 2016) could be used, alternatively also allowing to include both prosody and syntax within a single statistical model.

At the semantic level, as sentences unfold in a word-by-word manner, making semantic predictions of a word based on its prior context is equal to having some degree of certainty about the future (i.e., predictive coding). When instead an improbable event occurs, the prediction turns out to be an error. In computational approaches, the probability at which a listener is able to predict the meaning of the next word of the narrative from the preceding passage is measured by word-level entropy, and the corresponding prediction error when encountered a word is parameterized as word-level surprisal. Essentially, both indices can be derived from the conditional probability of word forms as calculated by standard toolboxes (Stolcke, 2002; Roark et al., 2009; Frank et al., 2015; Willems et al., 2016). Word-level entropy and surprisal can then be regressed against power of band-pass filtered continuous EEG in the beta– and gamma-bands *via* time-resolved multiple regression or the mTRF (Crosse et al., 2016; Ehinger and Dimigen, 2019; Sassenhagen, 2019). Individual beta coefficients would be then directly compared between patients and healthy controls, revealing if semantic prediction or prediction error are impaired in schizophrenia. In addition, the impact of major schizophrenia symptoms (e.g., auditory hallucinations) on semantic-level predictive coding could be evaluated *via* a comparison between patients with or without auditory hallucinations.

Importantly, group differences in the respective correlations between entropy/surprisal and beta–gamma-band power would readily define candidate frequencies and time windows to address the hypothesis of abnormal phase–amplitude coupling between the beta and gamma bands in schizophrenia (e.g., Bastos and Schoffelen, 2015; Hyafil et al., 2015). While translational application of the naturalistic approach has been initially employed in autism research (Brennan et al., 2018), its value for schizophrenia research awaits validation.

## Conclusion

We have outlined the potential of studying neural tracking in the functional characterization of linguistic deficits in schizophrenia. In our view, two threads should be followed: First, deficient theta-band tracking of syllables should be assessed as part of the “routine ERP analyses” in schizophrenia. While the exact mechanisms of theta-band tracking are still being debated, it is clear that it reflects both bottom-up and top-down mechanisms that might be altered in patients. Second, the previously proposed relationship between delta-band oscillations, prosody, syntax, and temporal prediction may help to study the corresponding deficits in schizophrenia in a hypothesis-driven manner, with the potential to dissociate underlying electrophysiological dysfunction(s). Third, the general role of the beta–gamma interplay in the generation and evaluation of predictions may be fruitful in elucidating the electrophysiological dysfunction(s) that correspond to contextual–semantic symptoms. While both threads connect well with the literature, the direct link between frequency bands and linguistic dysfunctions may be overly simplistic. While often neural oscillations are assigned to specific functions or oscillatory deficits are linked to specific deficits, we believe that since these are coupled across both spatial and temporal scales, they should be evaluated in unison in relation to the naturalistic paradigms we propose.

## Data Availability Statement

The original contributions presented in the study are included in the article/supplementary material, further inquiries can be directed to the corresponding author/s.

## Author Contributions

LM, PL, and YH wrote the manuscript. All authors contributed to the article and approved the submitted version.

## Conflict of Interest

The authors declare that the research was conducted in the absence of any commercial or financial relationships that could be construed as a potential conflict of interest.

## Footnotes

1 Note that our manuscript is restricted to hypotheses on auditory processes genuine to speech processing, as well as on higher-level linguistic processes. The independent modulation of these processes by attention and working memory, both of which are impaired in schizophrenia (Braff, 1993; Carter et al., 1998), is beyond the scope of the current article.
